# The effect of dietary transition on infant microbiota composition and metabolic activity captured with the simulator of the human intestinal microbial ecosystem (SHIME)

**DOI:** 10.1017/gmb.2025.10007

**Published:** 2025-06-13

**Authors:** Shadi Pakroo, Samira Soltani, Armin Tarrah, Gisèle LaPointe

**Affiliations:** Canadian Research Institute for Food Safety, Department of Food Science, University of Guelph, Guelph, ON, Canada

**Keywords:** infant, adult, gut microbiota, dietary transition, short-chain fatty acids

## Abstract

The Simulator of the Human Intestinal Microbial Ecosystem (SHIME) system was provided with baby feed for one week to stabilise the microbial community, followed by a 10-day period with baby feed and another 10-day period with adult feed. The study was conducted using sterilised and standardised feed formulations, which model dietary conditions in vitro. Following the transition from baby to adult feed, a significant reduction in the proportion of butyrate in comparison to total SCFA was found after transitioning to adult feed in both the transverse colon and distal colon bioreactors. Our findings suggest that abrupt early-life dietary changes from simple to complex carbohydrates as well as the exclusion of bovine milk proteins can transiently lower the ability of the microbiota to produce butyrate. The lack of additional microbial input leads to a delay or impairment of the adaptation to the modified feed composition. However, given the short treatment duration and sterilised feed composition, these findings should be interpreted within the limitations of this in vitro model. A reduction in butyrate concentration following the transition to adult feed may reflect a temporary shift in microbial metabolic activity rather than a long-term impact on energy extraction efficiency in vivo.

## Introduction

The neonatal gut microbiota rapidly acquires a diversity of bacteria from the mother, which then undergoes selection and succession as anaerobic conditions develop and oxygen is depleted (Hill et al., 2017; Ferretti et al., 2018). Subsequently, Actinomycetota, such as *Bifidobacterium*, become more predominant from 1 week to 14 months of age, primarily reflecting the level of milk consumption and breastfeeding in comparison to infant formula feeding (Stewart et al., 2018). As infants transition to a higher proportion of solid foods and reduce their consumption of milk, their gut microbiota diversity increases remarkably between 15 and 30 months (Stewart et al., 2018).

This period is characterised by the emergence of additional genera belonging to the Bacteroidota phylum, which produce short-chain fatty acids (SCFAs), such as acetate, and members of the Bacillota phylum, such as *Blautia* from the *Lachnospiraceae* family, which produce acetate and butyrate. These SCFAs help maintain gut integrity, support immune function, and provide energy to colon cells (Holmberg et al., 2024). By around 31 months, the microbiota diversity stabilises, marked by higher levels of Bacillota (e.g., *Faecalibacterium* and *Roseburia*) (Hill et al., 2017; Stewart et al., 2018).

Emerging evidence suggests that dietary transitions during infancy significantly influence the composition and diversity of the gut microbiota, thereby shaping host immune function, metabolism, and overall health outcomes through the acquisition of environmental microbes and the production of SCFAs via microbial fermentation of dietary fibres (Davis et al., 2020). Compared to the limited range of nutrients in breast milk, the introduction of early solid foods provides a broader array of nutrients, including diverse sources of fats, proteins, carbohydrates, and fibres, as well as exposure to additional microbes. These changes promote the diversification of the gut microbiota towards a more adult-like composition (Homann et al., 2021). Dietary components serve as substrates for microbial fermentation in the gut, leading to the production of metabolites, including the SCFAs acetate, propionate, and butyrate. These SCFAs play essential roles in regulating host physiology, immune responses, and energy metabolism (Afzaal et al., 2022; Di Profio et al., 2022). These dietary transitions, such as the reduction in milk consumption, the introduction of solid food, and the increased diversity of complex plant-derived carbohydrates during weaning, influence SCFA production through the succession of butyrate-producing microbes (Appert et al., 2020). Although many host-related factors are best investigated through *in vivo* studies, *in vitro* models are more suitable for determining the impact of specific nutritional interventions in each section of the gastrointestinal tract (Van den Abbeele et al., 2021), in the absence of environmental sources of microbes.

The aim of this study was to determine the rate of shift in the infant gut microbiota composition and the resulting metabolite balance during a dietary transition representing the reduction of milk consumption and an increase in complex carbohydrates. By using the Simulator of the Human Intestinal Microbial Ecosystem (SHIME), the effects of the host immune system, absorption of SCFAs, and other host-dependent factors can be subtracted (Van den Abbeele et al., 2021) to reveal the process of adaptation of the endogenous microbial community in response to the consecutive conditions of the colon.

## Methods

### Twin SHIME model

**Figure 1.**
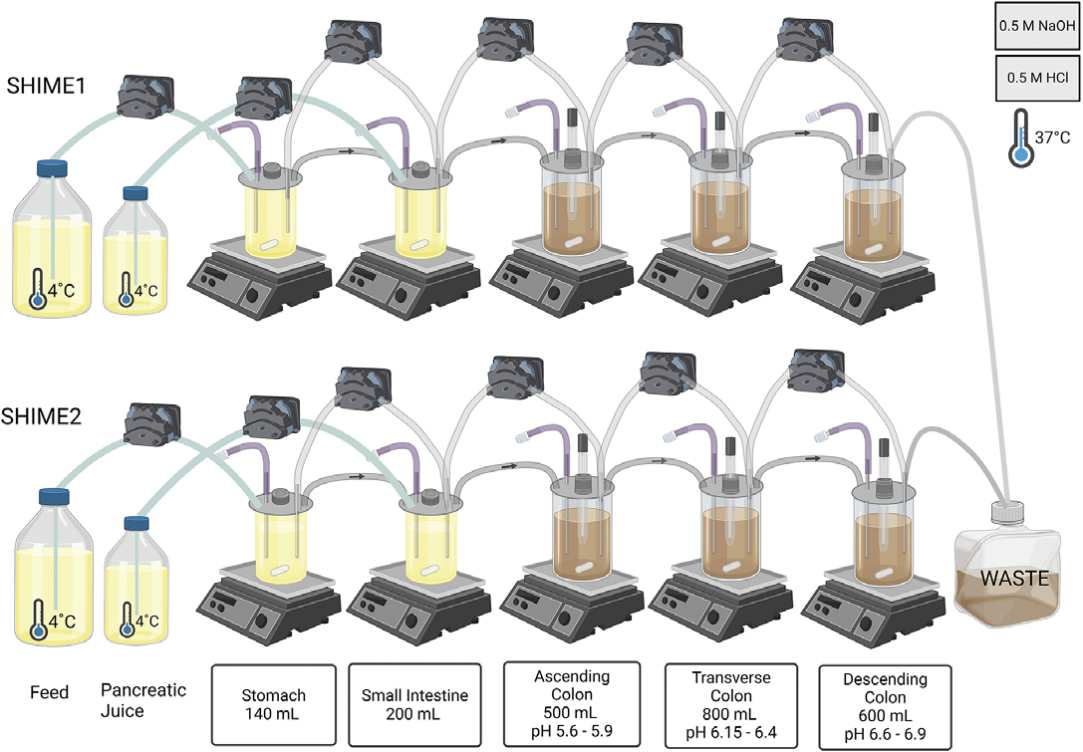
Overview of the Twin SHIME® system utilised in the study showing the setup, and configuration of the SHIME® system, including the compartments and associated conditions used for simulating the human gastrointestinal tract. In this study, SHIME® 1 and SHIME® 2 functioned as replicates undergoing the same series of treatments.

The Twin SHIME system was set up, adapted, validated, and operated in accordance with the SHIME manual (ProDigest-Ghent University, Belgium). The Twin SHIME system consisted of 10 vessels, configured as 2 parallel series of 5 vessels (Figure 1), each encompassing a stomach, a small intestine, and 3 colon regions: ascending colon (AC) (volume = 500 mL, pH = 5.6–5.9), transverse colon (TC) (volume = 800 mL, pH = 6.15–6.4), and descending colon (DC) (volume = 600 mL, pH = 6.6–6.9). The temperature was controlled at 37°C using a water bath, and the pH was adjusted in each colon vessel with 0.5 M NaOH or HCl. In addition, the vessels were continuously stirred at 300 rpm for a duration of 3 weeks. SHIME 1 and 2 served as replicates receiving the exact same treatments (Van den Abbeele et al., 2012).

### Faecal sample collection, slurry preparation, and inoculation

An infant donor was selected based on specific criteria to ensure a controlled and consistent baseline microbiota for this study (Mesnage et al., 2022). These criteria include an infant between 10 and 12 months of age who had not been introduced to solid foods and was exclusively fed breast milk and formula with a healthy infant microbiota composition, dominated by Actinomycetota, Bacillota, and Bacteroidota, which are the most abundant phyla in infants at this developmental stage (Jost et al., 2014; Hill et al., 2017; Derrien et al., 2019). The selected donor was a vaginally born, healthy, 11-month-old male who fit these criteria and had not received antibiotics for at least 3 months before sampling. Faecal samples were aseptically collected using sterilised containers. Subsequently, 20 g of the faecal sample was homogenised in 100 mL of 0.1 M phosphate buffer (20% w/v) using a stomacher at 200 rpm for 30 s (Deyaert et al., 2023). The resulting suspension was aliquoted into two 50 mL Falcon tubes and centrifuged at 500×*g* for 2 min. The supernatant was collected to obtain the final suspension. Each colon vessel was inoculated with faecal slurry at 5% AC (500 mL with 25 mL of inoculum), TC (800 mL with 40 mL of inoculum), and DC (600 mL with 30 mL of inoculum). Immediately after inoculation, automatic pH control was initiated, and the culture was allowed to incubate overnight before the initiation of the feeding schedule for the 7-day stabilisation period and then the 10-day treatment period.

### Feed and pancreatic juice preparation and uptake

To prepare the feed, 17.2 g/L of medium (PD-NM005) for baby feed and 15.6 g/L of L-SHIME medium (PD-NM001B) for adult feed were added to 1 L of distilled water (Table 1). After thorough mixing, the medium was autoclaved, and the pH was adjusted according to the ProDigest guidelines. The resulting feed was stored at 4°C and continuously stirred at 300 rpm throughout the entire SHIME run. The feed volume was 140 mL three times a day at 8-h intervals, with pumping initiated at 5:00 PM, 1:00 AM, and 9:00 AM into the two stomach vessels. The pancreatic juice was prepared by dissolving NaHCO_3_ (12.5 g/L, Anachemia, VWR International, 470302-440); Ox bile (6 g/L, Difco™, BD Biosciences, DF0128-17-8); and pancreatin (0.9 g/L, Alfa Aesar, Thermo Scientific, J19880.28) in distilled water, using a heater for thorough mixing. The resulting solution was stored at 4°C and continuously stirred at 300 rpm throughout the entire SHIME run. Pancreatic juice was added to the small intestine vessels at a concentration of 60 mL per feeding session three times a day. Pumping was initiated at 6:30 PM, 2:30 AM, and 10:30 AM. At the end of the 10-day feed treatment period, luminal samples were collected from AC1, AC2, TC1, TC2, DC1, and DC2 for DNA extraction, 16S ribosomal RNA (rRNA) gene amplicon sequencing, and SCFA analysis.

**Table 1.** Composition of the baby and adult feeds used in the experiment

Baby (PD-NM005)	Adult (PD-NM001B)
Pectin (1 g/L)	Pectin (2 g/L)
Glucose (1 g/L)	Glucose (0.4 g/L)
Mucin (6 g/L)	Mucin (3 g/L)
L-cysteine-HCl (0.2 g/L)	L-cysteine-HCl (0.5 g/L)
Starch (1 g/L)	Starch (4 g/L)
—	Arabinogalactan (1.2 g/L)
—	Xylan (0.5 g/L)
—	Yeast extract (3 g/L)
—	Special peptone (1 g/L)
Cellobiose (1 g/L)	—
Proteose peptone (2 g/L)	—
Lactose (2.1 g/L)	—
Casein (0.2)	—
Whey proteins-lactalbumin (2.7 g/L)	—

### DNA extraction and 16S rRNA gene amplicon sequencing

Total volume of 1 mL of luminal samples was centrifuged at (10,000×*g*, 10 min, 4°C) to collect cell pellets. Subsequently, DNA extraction was carried out using the DNeasy PowerSoil Pro Kits (Qiagen, MD, USA) following the manufacturer’s protocol. The quantity and quality of the extracted DNA were assessed through fluorometry (Qubit 4, Invitrogen, Waltham, MA, USA) and spectrometry (NanoDrop 1000; Thermo Scientific, Waltham, MA, USA), respectively. The DNA samples underwent dilution to 10 ng/mL and were subsequently submitted to the Advanced Analysis Centre (AAC) at the University of Guelph, Guelph, Ontario, Canada, for sequencing of the V3–V4 regions of the 16S rRNA gene amplicon using the Illumina MiSeq platform (Illumina, San Diego, CA, USA). Amplification of the V3 and V4 regions (~460 bp) of the 16S rRNA gene was achieved utilising the following universal primers: forward primer: 5′-CCTACGGGNGGCWGCAG-3′ and reverse primer: 5′-GACTACHVGGGTATCTAATCC-3′ (Klindworth et al., 2013). Raw sequence reads underwent filtering using the Miseq Sequencer System software to eliminate low-quality sequences and were trimmed to eliminate adapter sequences. The resultant reads ranged up to 301 bases in length.

### SCFA quantification by ^1^H-nuclear magnetic resonance

Then, 5 mL of luminal samples were subjected to filtration using a double-layer polystyrene filtration system in two steps: first with a 0.84-μm pore size filter, then with a 0.22-μm pore size filter, and subsequently stored at –20°C. Following thawing, 540 μL of the filtered luminal samples were mixed with 60 μL of IS-2 Chenomx Internal Standard-DSS-d6 (deuterated 2,2-dimethyl-2-silapentane-5-sulfonate-d6 sodium salt (5 mM) and sodium azide (0.2% w/v) in 99.9% D_2_O) (Chenomx, 613150). This resulting mixture was then carefully transferred to a 5-mm thin-wall precision nuclear magnetic resonance (NMR) sample tube (Wilmad-LabGlass, NJ, USA, WG-1000-4) and submitted for analysis at the NMR Centre of the AAC (University of Guelph, Guelph, ON).

### Bioinformatics and data analysis

All sequence data were processed using the DADA2 pipeline through the MicrobiomeAnalyst 2.0 server (Lu et al., 2023b). This process encompassed filtering, denoising, merging read pairs, and the removal of chimaeras. Finally, the operational taxonomic units (OTUs) were generated based on the SILVA database version 138.1 (McLaren and Callahan, 2021). Two samples, one from an adult-fed vessel (AC1) and one from a baby vessel (AC1), were removed due to insufficient read counts, notably falling below 500 reads. The statistical analysis was performed on the MicrobiomeAnalyst 2.0 server. Sequences with counts lower than 4 and a prevalence below 20% were filtered out. Following this, data normalisation was performed using total sum scaling, and the data were subsequently rarefied to a minimum library size of 40,662 sequences. This step aimed to alleviate any potential biases arising from differences in sequencing depth among various samples. Alpha diversity was assessed using the Shannon index, comparing the adult-fed and baby-fed groups, as well as among the AC, TC, and DC groups, using Welch’s *t*-test for pairwise comparisons and analysis of variance (ANOVA) for multiple group comparisons. For β-diversity analysis, principal coordinate analysis with the Bray–Curtis index was employed, followed by permutational multivariate ANOVA (PERMANOVA) to determine statistical significance among the groups. Principal component analysis (PCA) was conducted on microbial compositional data, including all genera above the filtering threshold, after *Z*-score normalisation to visualise shifts in microbial communities across feeds for the AC, TC, and DC vessels. Linear discriminant analysis effect size (LEfSe) was then used to detect significant differences in bacterial abundance between the treatment groups.

## Results

The baby and adult feeds differed in terms of simple versus complex carbohydrates and the presence or absence of bovine milk proteins (Table 1). The baby feed, which was intended to simulate the diet of post-weaning infants, contained a higher level of glucose (1 g/L) and lactose (2.1 g/L), while the adult feed had higher concentrations of starch (4 g/L) and pectin (2 g/L), as well as arabinogalactan and xylan. In addition, the baby feed includes casein (0.2 g/L) and whey proteins (2.7 g/L), which were not present in the adult feed. Proteins were present in both feeds, with the baby feed containing proteose peptone (2 g/L), casein, and whey, whereas the adult feed included special peptone (1 g/L) and yeast extract (3 g/L). The total carbohydrates in the baby feed amounted to 6.1 g/L, compared to 8.1 g/L in the adult feed. Conversely, the total protein content was slightly higher in the baby feed at 4.9 g/L compared to 4 g/L in the adult feed. As both feed types were autoclaved, no additional microbes were introduced into the stabilised microbial community over the period of study.

The two parallel SHIME systems, each comprising a stomach, small intestine, and three colon regions (AC, TC, and DC), were operated for 1 week to allow a sufficient period of adaptation before starting the feed transition. Microbiota stabilisation was confirmed by measuring butyrate, propionate, and acetate levels on 2 separate days, spaced 3 days apart, during the initial 1-week period of baby feed administration. Consistent SCFA production over two sequential measurements confirmed the stability of the metabolic activity of the microbiota (Van de Wiele et al., 2015).

A total of 1,160,868 high-quality reads with an average read count of 105,553 per sample were obtained after removing low-quality and chimeric sequences. The examination of rarefaction curves alongside an average Good’s coverage index of 99.99 ± 0.001 suggests that the sequencing depth comprehensively captured the bacterial diversity present in all samples examined in this study. With the OTU table (Supplementary Material S1), 1571 OTUs were considered for analysis after removing features with a minimum count below 4 and those with <10% variance across samples. In summary, within the baby-fed group, Bacillota was the most prevalent phylum at 37.7%, followed by Actinomycetota at 33.7% and Bacteroidota at 26.3%. In the adult-fed group, Actinomycetota emerged as the predominant phylum at 32.6%, followed by Pseudomonadota at 24.6% and Bacteroidota at 23.1%.

**Figure 2.**
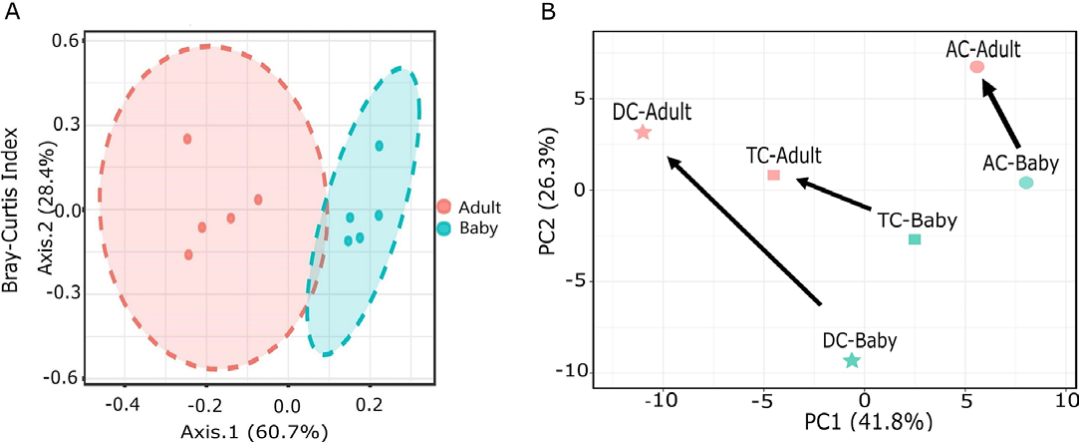
(A) Principal coordinate analysis (PCoA) based on the Bray–Curtis similarity index, illustrating beta diversity differences between baby and adult feeds. (B) Principal component analysis (PCA) illustrating microbial compositional shifts, with arrows indicating the transition from baby to adult feeding for each vessel.

**Figure 3.**
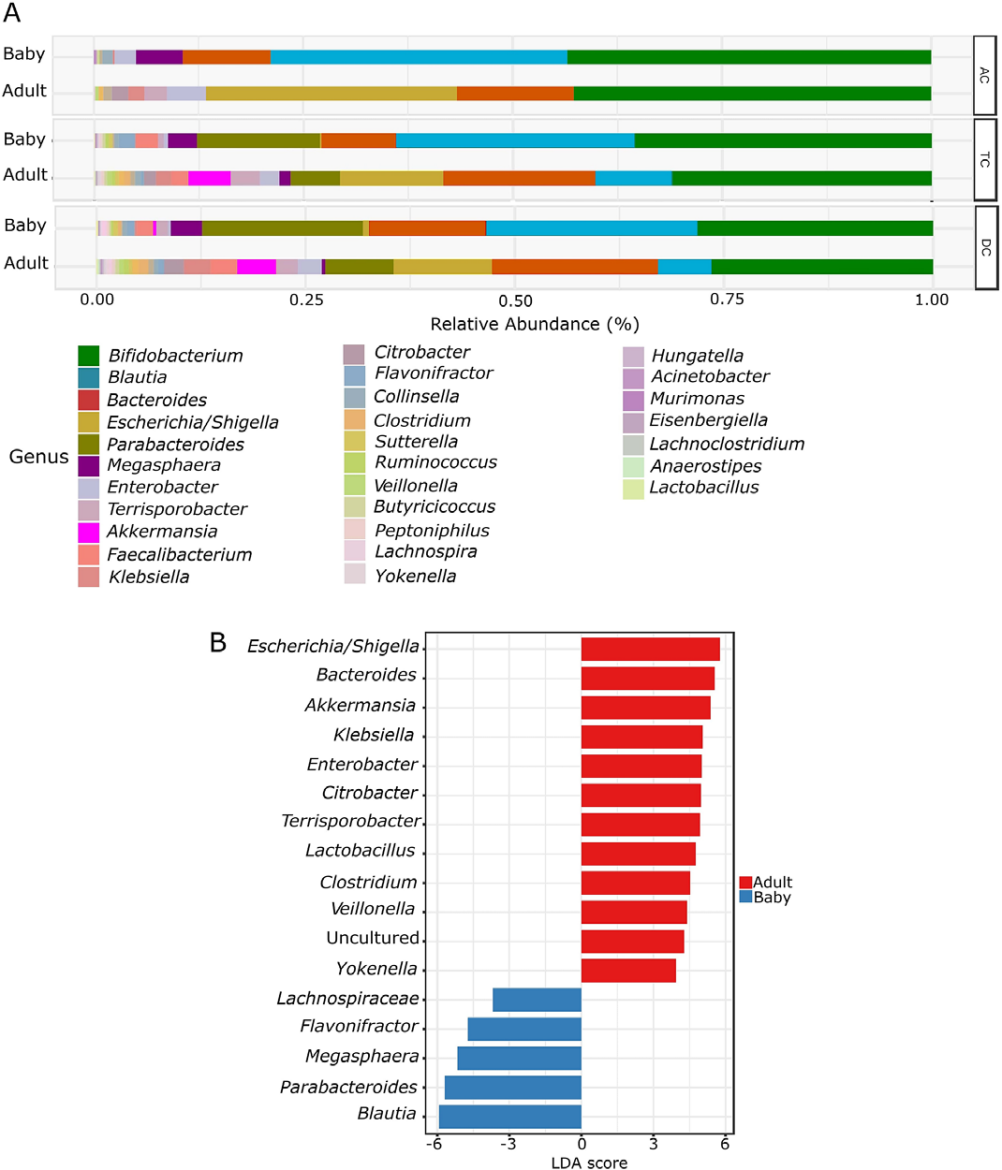
(A) Relative abundance of bacteria at the genus level among the feed types and colon sites. (B) Linear discriminant analysis effect size (LEfSe) at the genus level between the feed types. An LDA score > 2 was used to determine significantly different genera between the groups.

The alpha diversity did not differ significantly between the adult-fed and baby-fed treatments, as measured with the Shannon index, which considers both species richness and evenness (*p*-value: 0.102). The beta diversity analysis, utilising the Bray–Curtis similarity index, with PERMANOVA as the statistical method, revealed significant differences (*p*-value: 0.008) in microbial diversity between the baby-fed and adult-fed vessels (Figure 2A). PCA revealed a clear shift in microbial composition between baby-fed and adult-fed conditions across AC, TC, and DC colon sites (Figure 2B). This shift was consistent in direction across the AC, TC, and DC, highlighting a structured microbial transition within the conditions of the three vessels. *Bifidobacterium* was found to be the predominant genus in both baby-fed and adult-fed groups across all colon sites (AC, TC, and DC) (Figure 3A). LEfSe analysis revealed significant differences in genera among the treatment groups in all three colon sites (Figure 3B). Following the transition to adult feed, several genera were significantly reduced, including *Blautia*, *Parabacteroides*, *Megasphaera*, and *Flavonifractor.* Conversely, other genera, such as *Escherichia–Shigella*, *Klebsiella*, *Bacteroides*, *Akkermansia*, *Enterobacter*, *Citrobacter*, *Clostridium*, *Lactobacillus*, *Yokenella*, and *Veillonella*, were significantly higher in the adult-fed vessels.

**Figure 4.**
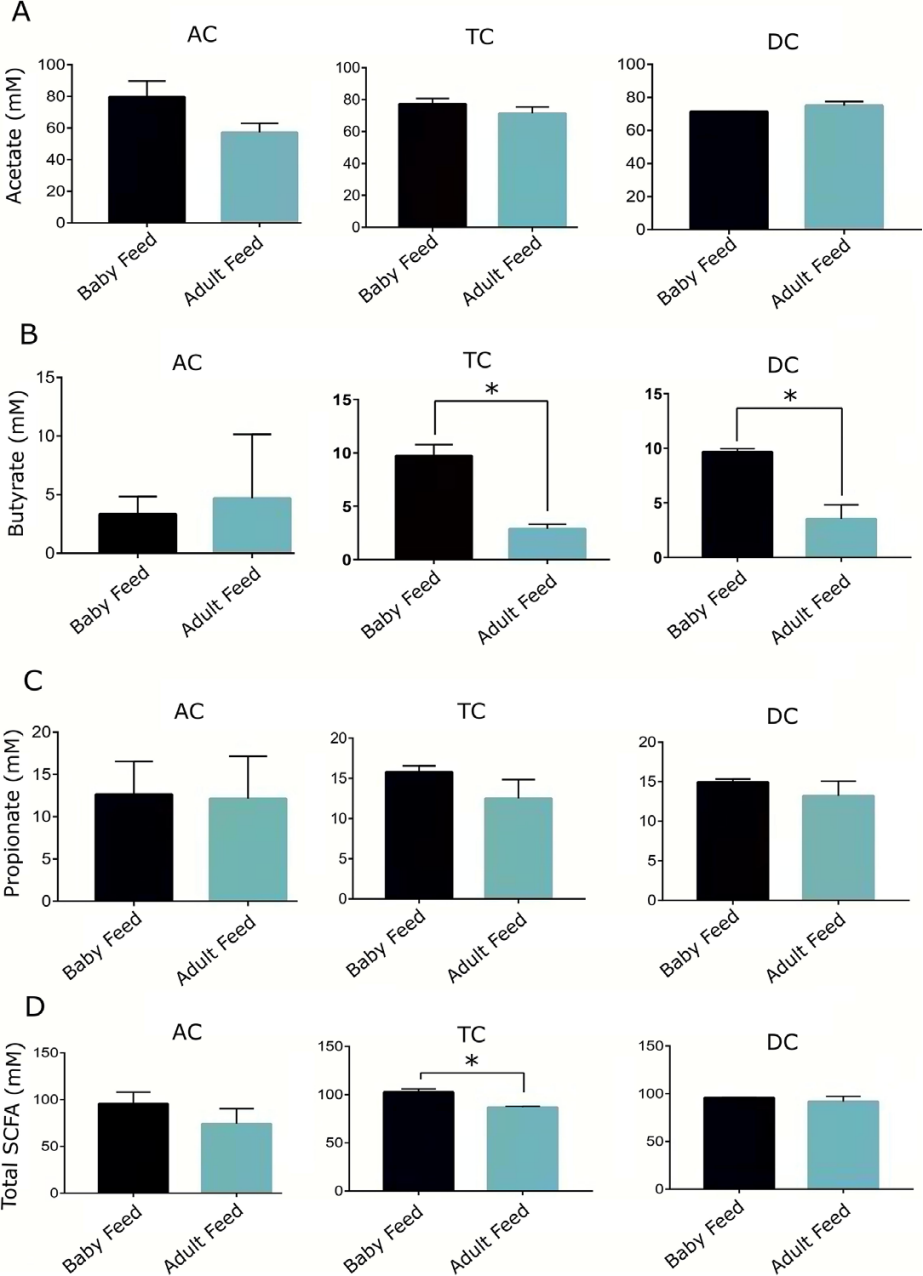
Concentration of acetate, butyrate, propionate, and total SCFA in the AC, TC, and DC vessels of the TwinSHIME system fed with baby and adult feed.

**Figure 5.**
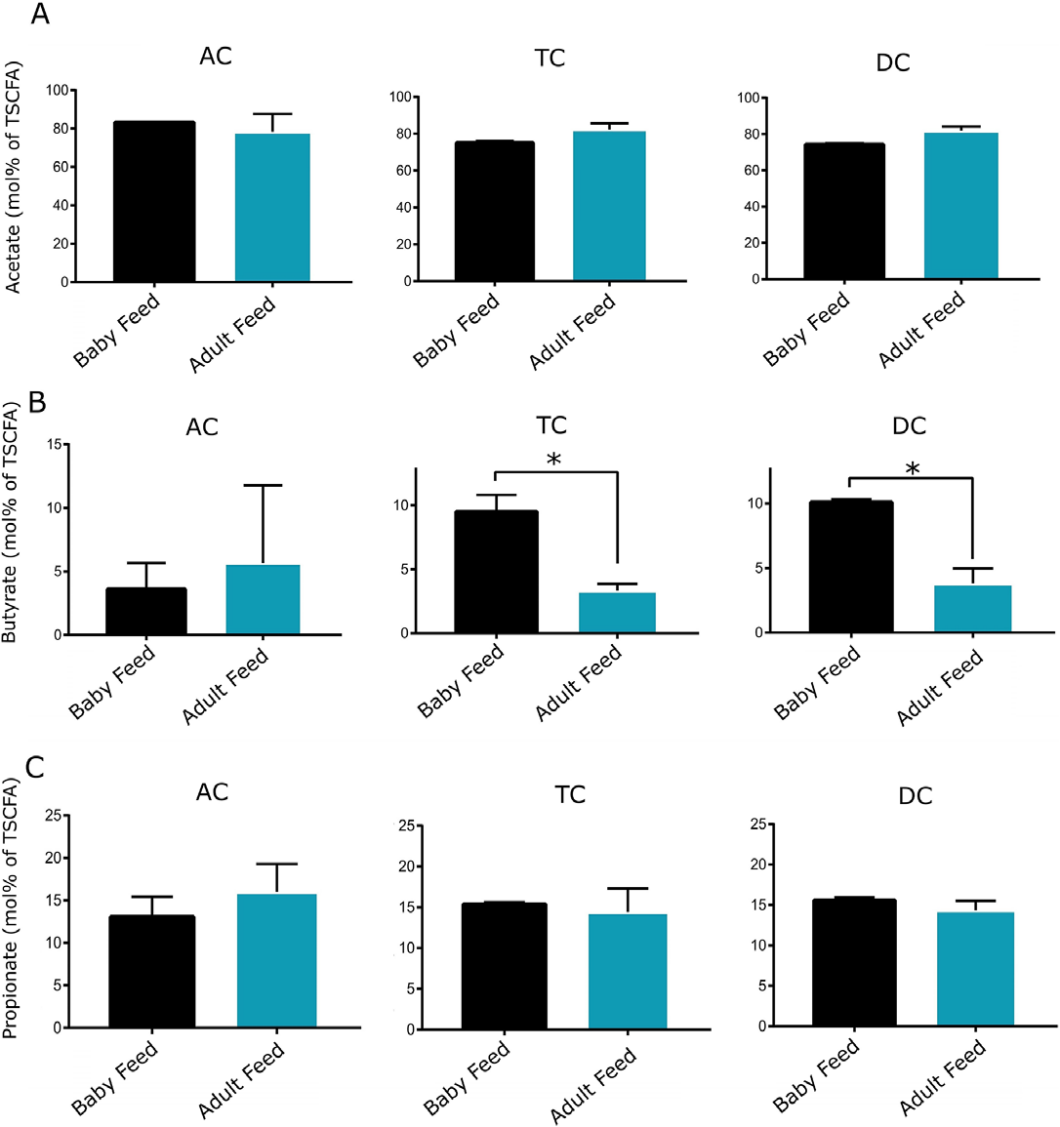
Molar proportion of acetate, butyrate, and propionate in the AC, TC, and DC vessels of the TwinSHIME system fed with baby and adult feed.

Regarding metabolic activity, the amount of total SCFA was significantly different between baby feed and adult feed treatments only for the TC, but not for the AC or DC regions (Figure 4). No significant differences were detected in acetate and propionate amounts or molar ratios between baby-fed and adult-fed vessels across all colon sites (Figures 4 and 5). However, a significant reduction in butyrate amount and molar ratio was observed after transitioning from baby feed to adult feed in both the TC and DC regions (two-tailed *t*-test, *p*-value < 0.05), which was not observed in the AC vessel.

## Discussion

In this study, the Twin SHIME® system was used to correlate changes in the gut microbiota composition with metabolic activity during the transition from baby feed to adult feed in conditions mimicking the three sections of the colon. The SHIME was employed to simulate infant gut microbiota under standardised conditions, minimising interindividual differences caused by variations in diet, pH, transit time, and other factors (Šuligoj et al., 2020). Over the stabilisation period, the SHIME establishes a steady-state microbial community, controlled by reproducible factors, such as feed content, pH, enzymes, and transit time (Šuligoj et al., 2020). This model also allows for a longitudinal study design, enabling the comparison of treatment effects with both the initial baseline and a concurrently run control (Van den Abbeele et al., 2021).

The higher content of simple carbohydrates, including glucose and lactose, in the baby feed supports the growth of Bacillota populations, which primarily metabolise these easily fermentable carbohydrates (Gong et al., 2020). Specifically, Bacillota contribute significantly to the gene repertoire of glycoside hydrolases and carbohydrate esterases (Gong et al., 2020). Bacillota play a role in the human gut by fermenting carbohydrates, such as glucose and lactose, to produce SCFAs, such as acetate, propionate, and butyrate, which are important for gut health and energy metabolism (Desai and Landay, 2018; Baltazar-Díaz et al., 2024).

*Blautia*, *Parabacteroides*, and *Megasphaera* genera were more abundant in the microbial communities under the baby feed. Studies have shown that *Blautia* species thrive in environments rich in short-chain fructooligosaccharides (Mao et al., 2018) and lactulose (Cui et al., 2021), and are known for producing butyrate via the acetyl-CoA pathway (Zhou et al., 2017). Furthermore, lactose, a major carbohydrate in ProDigest baby feed, supports the growth of bacteria such as *Bifidobacterium* that are well-known for their ability to metabolise this disaccharide. The lactate produced from lactose or xylose fermentation by these bacteria can then be utilised by *Megasphaera*, which converts lactate into butyrate, which is an SCFA essential for maintaining gut health (Zhao et al., 2024). This cross-feeding interaction provides *Megasphaera* with a competitive advantage by allowing it to increase in environments where lactose or xylose are present, thereby contributing to a beneficial gut environment (Shetty et al., 2013). Previous research has shown that a lower abundance of *Megasphaera* as a biomarker of gut health in infants is associated with an increased risk of diarrheal symptoms in cryptosporidiosis (Carey et al., 2021). The genus *Parabacteroides* includes several species with a high number of mucin-degrading carbohydrate-active gene clusters per genome, which facilitates their proliferation in environments containing mucin (Pruss et al., 2021). In our study, the baby feed contained a higher mucin concentration of 6 g/L compared to the adult feed, which contained 3 g/L mucin. This higher mucin content in the baby feed provided a favourable substrate for the growth of *Parabacteroides* strains, which are specialised in degrading mucin effectively (Pan et al., 2022). These mucin-degrading gut commensals, particularly *Parabacteroides* spp., exhibit anti-inflammatory properties and enhance the epithelial barrier (Pan et al., 2022). *Akkermansia* is also known for its ability to degrade mucin and is supported by the presence of mucin in both baby and adult feeds (Fricker et al., 2024). Although the mucin concentration in the baby feed was higher (6 g/L) compared to the adult feed (3 g/L), *Akkermansia* showed increased abundance during the adult feed period. This suggests that other factors, such as cross-feeding from complex carbohydrates or proteins present in the adult feed, may have contributed to the higher relative abundance of *Akkermansia*, indicating that the metabolism of the complex carbohydrates in the adult feed created a favourable environment for its growth (Belzer and De Vos, 2012). The combination of *Bifidobacterium* and prebiotic fibres has been shown to promote the abundance of *Akkermansia* (Zhang et al., 2022b).

The adult feed contains a higher concentration of complex carbohydrates, such as pectin, starch, arabinogalactan, and xylan. Bacteroidota and Actinomycetota contribute to the digestion of starch and complex polysaccharides, such as pectin, arabinogalactan, and xylan, breaking them down into SCFAs, such as acetate and propionate that further support gut function (Vinke et al., 2017; Fujita et al., 2019; Rinninella et al., 2019). Typically, *Bacteroides* produces more acetate than propionate. In our study, acetate was dominant in the overall SCFA profile observed, coinciding with the higher abundance of *Escherichia–Shigella* and *Bacteroides* (Cockburn and Koropatkin, 2016; Zhang et al., 2022a). However, neither the amount nor the molar proportion of acetate differed between the two feeds. The presence of both fibre-degrading and carbohydrate-utilising bacteria determines the equilibrium of SCFA metabolites (Flint et al., 2012). *Bifidobacterium* was found in both baby-fed and adult-fed groups across all colon sites. Bifidobacterial species tend to dominate the intestines of healthy breastfed infants, but the dominant species change with age, generally decreasing in adulthood and stabilising at lower levels (Arboleya et al., 2016), while its abundance can vary significantly between individuals (Lu et al., 2023a). The presence of lactose in baby feed explains the higher abundance of bifidobacteria observed during the baby feed period. As the diet transitions from baby feed to more complex adult feed, the presence of *Bifidobacterium* reflects the ability to degrade arabinogalactan and xylan substrates, producing acetate. This suggests that *Bifidobacterium* can be carried over into the adult period, maintaining acetate production in the absence of lactose, but at a lower relative abundance.

The stability of taxa such as Actinomycetota, which showed minimal changes in relative abundance between the baby-fed and adult-fed periods, shows the resilience of certain microbial populations during dietary shifts. Despite the dietary shift towards complex carbohydrates, which supports the proliferation of *Bacteroides*, *Bifidobacterium* maintains a stable presence, partly due to syntrophic interactions with *Bacteroides* (Munoz et al., 2020). These interactions are particularly evident in the degradation of arabinogalactan, a complex polysaccharide, where *Bacteroides* initially break down the polysaccharide into oligosaccharides and other intermediates, some of which are released into the surrounding environment. *Bifidobacterium* can then utilise these partially degraded products through cross-feeding, thus establishing a cooperative metabolic network (Munoz et al., 2020). However, the transition from a baby feed rich in simple sugars and oligosaccharides to an adult feed containing more complex carbohydrates raises the possibility that *Bifidobacterium* populations may decline over time due to the reduced availability of their preferred substrates (Odamaki et al., 2016). Given that *Bifidobacterium* prefer simpler sugars, such as lactose, and are also adept at transporting and utilising a variety of oligosaccharides, including human milk oligosaccharides and prebiotics, their abundance may eventually decline in the absence of adequate fermentable substrates. These substrates are more prominent in baby diets, which could explain why *Bifidobacterium* populations may be less stable as the diet shifts to complex carbohydrates in adulthood. The timing of this decline could depend on how quickly other microbial populations, such as *Bacteroides*, dominate the ecological niche as they are better equipped to utilise the more complex carbohydrates present in the adult feed (Xiao et al., 2024).

*Escherichia–Shigella* were more abundant after the adult feed treatment, which was enriched in complex carbohydrates. *Escherichia–Shigella*, including commensal *Escherichia coli* species within the Pseudomonadota phylum, can utilise a range of monosaccharides and disaccharides derived from the breakdown of carbohydrates, producing acetate (Fabich et al., 2008). The *Escherichia* genus belongs to the Proteobacteria (Pseudomonadota) phylum and was found to increase in abundance during the transitional phase of the gut microbiota development of infants from 15 to 30 months of age (Stewart et al., 2018).

The milk proteins in baby feed, including both whey protein and casein, play a role in promoting Bacillota and Actinomycetota (Monteiro et al., 2016; Beaumont et al., 2017). Whey proteins have been observed to support the growth of *Bifidobacterium*, which belongs to the phylum Actinomycetota (Petschow and Talbott, 1990). Other bacteria also utilise whey proteins; however, studies indicate that whey protein more effectively stimulates the growth of *Bifidobacterium* compared to other protein sources, which is likely due to the specific adaptability of these bacteria to whey proteins, as they possess specialised enzymatic pathways, such as protease activity and peptide transport systems that enable them to efficiently metabolise whey proteins into acetate and lactate through amino acid-to-SCFA conversion pathways (Pescuma et al., 2010; Sánchez-Moya et al., 2017; Boscaini et al., 2023). The acetate and lactate produced can be further utilised by other gut bacteria, particularly butyrate-producing bacteria, through cross-feeding interactions, influencing butyrate production in the gut (Zhao et al., 2024). Some clostridia can convert amino acids such as glutamate to butyrate (Louis and Flint, 2017). In contrast to the protein sources found in baby feed, the adult feed used in the study incorporates peptones derived from animal and plant proteins (Table 1). The consumption of animal proteins has been associated with an increase in the abundance of Pseudomonadota in the gut microbiota (Zhu et al., 2015). In one study, rats fed meat proteins exhibited a higher prevalence of the phylum Pseudomonadota compared to those fed casein, while the group fed soy protein demonstrated a significant increase in Bacteroidota (Zhu et al., 2015). This finding aligns with the notable rise in the genera *Escherichia*–*Shigella*, *Klebsiella*, *Citrobacter*, *Enterobacter*, and *Bacteroides* observed in our adult feed group. Interestingly, the same study also reported an increase in the abundance of the genus *Lactobacillus* among those consuming animal proteins, especially chicken and fish, compared to the casein group (Zhu et al., 2015).

Acetate and propionate levels did not differ between the baby- and adult-fed treatments across all colon sites, even though the starch and complex carbohydrate levels were higher during the adult feeding period. Thus, the change in carbon substrates between feeds did not modify the relative proportions of acetate and propionate-producing bacteria, even though the specific groups of microbes were altered in abundance. However, both the amount and the molar ratio of butyrate declined in the TC and DC regions after transitioning to adult feed. Elevated relative abundance of *Escherichia*–*Shigella* is often associated with a lower abundance of SCFA-producing bacteria (Hu et al., 2022). This reduction in butyrate levels upon switching to adult feed could be attributed to the relative proportions of bacteria able to degrade arabinogalactan and xylan. Acetate-producing *Blautia* spp. can utilise starch but may not be able to degrade xylan (Chen et al., 2020) or arabinogalactan (Nie et al., 2023), explaining its decline under the adult feed. The stabilised microbiota composition in the SHIME model derived from the infant donor showed the presence of butyrate-producing bacteria, such as *Lachnospiraceae*, *Ruminococcaceae*, and *Clostridiaceae. Megasphaera*, which produces butyrate from organic acids, such as lactate, declined after the transition to adult feed but was maintained at a lower relative abundance. With this decline in both *Blautia* and *Megasphaera*, the combined relative abundance of minor components of the microbial community increased, such as *Clostridium* spp., which is considered one of the pioneering butyrate producers in the gut, but they produce low amounts of butyrate (Appert et al., 2020). Appert et al. (2020) have demonstrated that butyrate-producing groups are generally absent in infant faeces but increase in prevalence and abundance over the first year of life. Some of these species have limited ability to use complex carbohydrates, such as *Anaerobutyricum halli*, so they depend on cross-feeding, and converting lactate and acetate to butyrate. Many *Lachnospiraceae* can utilise a diversity of carbohydrates, generating larger amounts of butyrate (Appert et al., 2020). This implies that the relative ratio of *Lachnospiraceae*, *Ruminococcaceae*, *Erysipelotrichaceae*, and *Clostridiaceae* within the Bacillota group determines the rate of butyrate production. The decrease in butyrate in the adult-fed vessels is consistent with the decline in *Lachnospiraceae* (higher butyrate producers) and the significant increase in *Clostridium* (lower butyrate producers). As both the baby and adult feeds used in this protocol were sterilised, no additional source of environmental microbes was provided. This ensured that the contribution from the feed itself was eliminated, revealing the metabolic adaptability of the original microbial community from the donor. This result demonstrates how the environmental microbes consumed by infants after weaning contribute to the further development of the ability of the gut microbiota to metabolise complex carbohydrates and increase butyrate production over the first year of life (Appert et al., 2020).

*Akkermansia*, a mucin-degrading bacterium, was more abundant in the adult feed group and is known to produce both propionate and acetate. In our study, the reduction in butyrate levels could be attributed to the fact that the 10-day transition period may not have been long enough for butyrate-producing bacteria to fully adapt and establish themselves if they were initially present at low levels (Zhao et al., 2018). The SHIME system did not receive any exogenous microbes from feed, as would occur with a human infant. Complex carbohydrates can be fermented by a range of bacteria, some of which produce acetate or propionate rather than butyrate (Flint et al., 2012). Therefore, without sufficient butyrate-producing bacteria, the increased availability of complex carbohydrates might not result in higher butyrate production as expected (Cronin et al., 2021). While the modulation of SCFA production is well-documented, our findings suggest that during the transition to adult feed, if the prevalence of fibre-degrading bacteria is insufficient, butyrate production can decrease. As the feed was sterilised, the introduction of additional fibre-degrading microbes was limited, which may have inhibited or delayed the full development of the adult microbiota profile. The combination of an adult feed containing a defined set of carbohydrates and the lack of exogenous incoming bacteria limited the further diversification of the gut microbiota. This highlights the potential need for unsterilised feed or targeted microbial supplementation to reproduce the normal maturation of the microbial community to be able to efficiently produce butyrate.

As the concentration and percentage of acetate and propionate remained unchanged across the dietary transition, the functional capability of the gut microbiota was preserved despite changes in microbial taxa composition, supporting the resilience of the gut microbiota to this dietary transition. This suggests that dietary interventions focusing on promoting butyrate-producing bacteria may be advantageous to promote SCFA production during transitions such as weaning. The presence of adaptable bacterial taxa such as *Bacteroides*, *Escherichia*, and *Akkermansia*, contributes to the resilience of the gut microbiota, which can compensate for changes in other microbial populations while maintaining acetate and propionate production under varying dietary conditions (Wexler, 2007; Belzer and De Vos, 2012; Lozupone et al., 2012).

Our study showed the shifts in microbial species and some concomitant changes in SCFA production patterns that can be associated with the replacement of simple sugars with complex carbohydrates and the switch from milk proteins to meat and plant proteins representing the transition from a baby to adult feed. Notably, the significant reduction in butyrate production following the switch to adult feed illustrates the importance of the diet in providing additional fibre-degrading microbes to maintain butyrate production after the shift from simple sugars to complex carbohydrates. These findings suggest that maintaining or enhancing the abundance of butyrate producers during dietary transitions could be important for sustaining optimal energy extraction during development. While this study provides valuable insights into microbial shifts during dietary transitions, the findings are based on a single donor and should be interpreted within the specific context of this controlled study. The dietary intervention demonstrated the capacity of certain bacteria from the infant gut microbiota, such as *Akkermansia* and *Bacteroides*, to adapt to adult feed, but future interventions may need to include targeted probiotics, fibres, or prebiotics that specifically promote the growth of butyrate-producing bacteria to restore and enhance butyrate levels.

## Supporting information

Pakroo et al. supplementary material Pakroo et al. supplementary material

## Data Availability

The raw reads were deposited publicly in the Sequence Read Archive (SRA) database under the BioProject number “PRJNA1110247.”

## Supplementary material

The supplementary material for this article can be found at http://doi.org/10.1017/gmb.2025.10007.
